# Hyperchloremic metabolic acidosis due to saline absorption during laser enucleation of the prostate: a case report

**DOI:** 10.1186/s40981-022-00499-3

**Published:** 2022-03-10

**Authors:** Makiko Tabuchi, Kohei Morozumi, Yuichi Maki, Daisuke Toyoda, Yoshifumi Kotake

**Affiliations:** Department of Anesthesiology, Toho University Ohashi Medical Center, 2-22-36, Ohashi, Meguro, Tokyo, 153-8515 Japan

**Keywords:** Transurethral laser enucleation of the prostate, Saline, Hyperchloremia, Metabolic acidosis, Stewart approach

## Abstract

**Background:**

Recent technological advancements have enabled the use of electrolyte solutions such as saline or buffered electrolyte solution during transurethral resection or laser enucleation of the prostate. However, saline absorption may cause hyperchloremic metabolic acidosis.

**Case presentation:**

A male in his late seventies underwent holmium laser enucleation of the prostate under a combination of subarachnoid block and general anesthesia. Intraoperatively, abdominal distension prompted the attending anesthesiologist to consider the possibility of SGA malposition, and the trachea was intubated. Oropharyngeal and neck edema was observed, and laboratory examination revealed considerable acidosis with hyperchloremia. Further evaluation confirmed the absorption of a large amount of saline into the circulation via the perforated bladder. Application of the simplified Stewart approach clearly suggested that hyperchloremia was the principal cause of metabolic acidosis. The dilution of albumin attenuated acidosis.

**Conclusions:**

Absorption of normal saline during laser enucleation of prostate caused hyperchloremic metabolic acidosis and airway edema.

## Background

The absorption of non-electrolyte irrigation fluid during transurethral resection (TUR) of the prostate has been known to cause TUR syndrome [1, 2]. This syndrome is characterized by hyponatremia, hypo-osmolar state, brain edema, and temporal neurological deficits. However, recent technological advancements in transurethral surgery have enabled the use of normal saline in bipolar TUR and laser enucleation. In these procedures, a superior coagulation profile and the use of saline considerably reduces the volume, risk of irrigating fluid absorption, and electrolyte abnormalities [3–7]. Nonetheless, saline absorption during TUR may cause different pathophysiological changes from the conventional TUR syndrome. Actually, we found several cases of hyperchloremic acidosis after the absorption of large amount of normal saline during laser enuculeation of prostate and surgical hysteroscopy in the literature [8–12]. In this report, we describe a case with hyperchloremic metabolic acidosis and airway edema possibly caused by the absorption of normal saline during laser-assisted TUR of the prostate, wherein we applied the simplified Stewart approach to evaluate acid-base abnormalities [13].

## Case presentation

A male in his late seventies was scheduled to undergo holmium laser enucleation of the prostate (HOLEP) owing to benign prostate hypertrophy. He regularly consumed alcoholic beverages but was otherwise healthy. Since prolonged surgery was anticipated, the patient underwent subarachnoid block with hyperbaric bupivacaine, followed by general anesthesia; the airway was secured with a supraglottic airway (SGA).

Ninety minutes after the start of the HOLEP procedure, the anesthesiologist experienced progressive difficulty in ventilating this patient with increased airway pressure, poor end-tidal CO_2_ trace, and abdominal distention. These circumstances prompted the anesthesiologist to consider the possibility of SGA malposition and inadvertent insufflation of the stomach via a misplaced SGA. Therefore, the anesthesiologist intended to remove the SGA and re-intubate using a standard tracheal tube. During intubation, the provider found abnormally swollen oral and pharyngeal mucosa; nonetheless, intubation was successful. The presence of clinically relevant upper airway edema was confirmed by laryngofiberscopy performed by an otorhinolaryngologist immediately after intubation.

After intubation, arterial blood gas analysis revealed marked hyperchloremic metabolic acidosis (Table 1). Derived parameters such as NaCl base excess effect, albumin base excess effect, and lactate base excess effect are also presented in Table 1 [13].

**Table 1:** Perioperative blood gas and laboratory indices and evaluation of acid-base status using the simplified Stewart approach

	Preoperative	Intraoperative	ICU admission	First postoperative day
Hb (g/dl)	15.7	11.7	14.2	14.9
Albumin (g/dl)	3.9	2.2	2.9	2.5
Na (mmol/l)	142	145	145	143
Cl (mmol/l)	105	120	114	108
pH	n/a	7.217	7.236	7.358
HCO_3_^-^	n/a	16.7	17.6	20.8
Lactate (mmol/l)	n/a	0.8	0.9	2
NaCl base excess effect	2	-10	-4	0
Albumin base-excess effect	0.8	5	3.3	4.2
Lactate base-excess effect	n/a	0.2	0.1	−1

*n/a* not available

Hemoglobin, albumin, Na, and Cl levels were measured at the central laboratory, while other parameters were analyzed using a blood gas analyzer (Cobas B221, Roche Diagnostics, Basel, Switzerland). NaCl base excess effect, [Na^+^]-[Cl^-^]-35 (mEq/l); albumin base excess effect, 0.25X(42-Alb (g/l)) (mEq/l); lactate base excess effect, 1-Lactate (mmol/l) (mEq/l) (from reference [13])

Based on these findings, we suspected translocation of irrigating saline to the intravascular space from the peritoneal cavity through the perforated bladder. This hypothesis was supported by the large positive difference between the infused and recovered amounts of irrigating fluid and decreased hemoglobin and albumin levels without any major blood loss and was verified by fluid collection in the peritoneal space found by ultrasound examination. The balance between the used and recovered irrigation fluid was estimated to be +6400 ml.

Owing to concerns regarding airway compromise, the patient remained intubated and mechanically ventilated in the ICU until postoperative day 1. Diuresis with furosemide and carperitide during the ICU stay resulted in a negative fluid balance of 5200 ml and normalization of hyperchloremia (Table 1). The patient was transferred to the hospital ward without any sequel on the next day.

## Discussion

In this case, we assume absorption of normal saline during laser enucleation of prostate caused hyperchloremic metabolic acidosis and airway edema. We found two relevant publications about this complication. Grove et al. reported a case with pulmonary, facial, and upper-arm edema during hysteroscopy under general anesthesia using lactate Ringer solution as irrigating fluid; the authors found extensive hemodilution and concluded that the absorption of lactate Ringer caused hypervolemia as well as pulmonary and peripheral edema [8]. Dodd et al. also reported a case of hemodilution, facial and neck edema, and hyperchloremic metabolic acidosis during HOLEP wherein saline was used [9]. These cases supposedly share the same pathophysiological process although the mechanism of absorption and type of irrigating fluid were different from that in our case. Typical TUR syndrome is caused by the expansion of intracellular space due to the hypo-osmotic nature of non-electrolyte irrigating fluid. Instead, the iso-osmotic solution is principally distributed to the extracellular space and its absorption is suspected to cause expansion of the intravascular and extracellular spaces without affecting the intracellular space [14]. Thus, the extensive hemodilution and airway edema found in these cases may be the consequences of this expansion intravascular and extracellular space.

The underlying mechanism of metabolic acidosis was rather obvious owing to the marked hyperchloremia [15]. Physiochemical approach developed by Stewart has several advantages over traditional bicarbonate centered approach to interpret hyperchloremic metabolic acidosis [16, 17]. However, standard Stewart approach requires the measurement of total protein and phosphate. Additionally, quantitative and trend assessment of acid-base disorder is somewhat complicated. In this report, we applied the simplified Stewart approach to evaluate acid-base change [13]. In this approach, three factors are used to evaluate in base excess and expressed as equivalent to conventional base excess: the NaCl effect, albumin effect, and lactate effect. The primary cause of metabolic acidosis of this case is typified by the increase of the NaCl effect. Hypoalbuminemia caused by hemodilution partially countered the NaCl effect, and the lactate effect was negligible. The effects of diuretic treatment such as normalization of chloride and albumin during the ICU stay are easily appreciated by the change of these parameters. We believe that semi-quantitative assessment with the simplified Stewart approach is useful for the diagnosis and treatment of metabolic acidosis.

In conclusion, this case report documents the hyperchloremic metabolic acidosis and upper airway edema possibly caused by saline absorption during the transurethral procedure. Absorption of iso-osmotic fluid such as saline is characterized by the expansion of extracellular space and accompanying facial and airway edema. Application of the simplified Stewart approach was useful for interpreting the cause of metabolic acidosis.

## Data Availability

The datasets analyzed in this study are included in this published article.

## Abbreviations

HOLEP: Holmium laser enucleation of prostate
SGA: Supraglottic airway
TUR: Transurethral resection
